# Controlling Thermal Expansion Behaviors of Fence-Like Metal-Organic Frameworks by Varying/Mixing Metal Ions

**DOI:** 10.3389/fchem.2018.00306

**Published:** 2018-07-24

**Authors:** Hao-Long Zhou, Jie-Peng Zhang, Xiao-Ming Chen

**Affiliations:** ^1^MOE Key Laboratory of Bioinorganic and Synthetic Chemistry, School of Chemistry, Sun Yat-Sen University, Guangzhou, China; ^2^Department of Chemistry and Key Laboratory for Preparation and Application of Ordered Structural Materials of Guangdong Province, Shantou University, Shantou, China

**Keywords:** thermal expansion, porous coordination polymers, metal-organic framework, flexibility, pyridyl-carboxylate, structure-property relationship, metal ion radius, solid solution

## Abstract

Solvothermal reactions of 3-(4-pyridyl)-benzoic acid (Hpba) with a series of transition metal ions yielded isostructral metal-organic frameworks [M(pba)_2_]·2DMA (MCF-52; M = Ni^2+^, Co^2+^, Zn^2+^, Cd^2+^, or mixed Zn^2+^/Cd^2+^; DMA = *N*,*N*-dimethylacetamide) possessing two-dimensional fence-like coordination networks based on mononuclear 4-connected metal nodes and 2-connected organic ligands. Variable-temperature single-crystal X-ray diffraction studies of these materials revealed huge positive and negative thermal expansions with |α| > 150 × 10^−6^ K^−1^, in which the larger metal ions give the larger thermal expansion coefficients, because the increased space not only enhance the ligand vibrational motion and hinged-fence effect, but also allow larger changes of steric hindrance between the layers. In addition, the solid-solution crystal with mixed metal ions further validates the abundant thermal expansion mechanisms of these metal-organic layers.

## Introduction

Most solids expand slightly as temperature increases (0 < α < 20 × 10^−6^ K^−1^, α for axial thermal expansion coefficients, α = ∂*l*/∂*T* × 1/*l*), which is known as thermal expansion or positive thermal expansion (PTE). Though the structure changes of PTE materials are very small, thermal expansion can often affect other material properties, for example, lead to the loss of precision and function for optical instruments, microelectronic devices so on (Evans, 1999; Liu et al., 2018). In contrast, materials with abnormal thermal expansion behaviors, such as zero thermal expansion (ZTE, |α| ≈ 0 × 10^−6^ K^−1^), negative thermal expansion (NTE, α < 0 × 10^−6^ K^−1^), or very large thermal expansion (|α| > 100 × 10^−6^ K^−1^), are scarce (Mary et al., 1996; Chapman et al., 2005; Goodwin et al., 2008; Das et al., 2010; Zhou et al., 2015). NTE compounds may be applied to compensate the thermal expansion of a “normal” PTE material, so that it can remain functional in extremely high or low temperatures without degradation (Mary et al., 1996; Chapman et al., 2005; Rowsell et al., 2005; Wu et al., 2008; Zhou et al., 2016). On the other hand, materials with exceptionally large PTE and NTE can be useful to design sensitive thermomechanical actuators (Das et al., 2010; Zhou et al., 2013, 2015). However, designing and controlling thermal expansion behaviors of materials are still great challenges.

By virtue of their notable porosities and framework flexibilities, porous coordination polymers (PCPs), also known as metal-organic frameworks (MOFs), can show large structural responses toward the change of various environmental parameters, such as temperature, pressure, and magnetic/electric fields (Férey and Serre, 2009; Horike et al., 2009; Nagarkar et al., 2014; Schneemann et al., 2014; Chang et al., 2015; Kanoo et al., 2017; Zhang et al., 2017). Researches on the abnormal thermal expansion behaviors of MOFs have received more and more attentions in recent years (Rowsell et al., 2005; Yang et al., 2009; DeVries et al., 2011; Grobler et al., 2013; Wei et al., 2013; Zhou et al., 2013, 2015; Cai and Katrusiak, 2014; Li et al., 2017). Due to the unique host-guest interactions, the thermal expansion of MOFs can be effectively tuned by the type and amount of guest molecules (Phillips et al., 2008; Yang et al., 2009; Grobler et al., 2013). Besides the steric hindrance and supramolecular interactions caused by the guest molecule (Goodwin et al., 2005; Grobler et al., 2013), the motion of a guest molecule and the thermal expansion of a guest aggregation can also modulate the thermal expansion properties of framework materials (Zhou et al., 2013, 2015). However, the thermal expansion coefficients of MOFs are difficult to tune finely and on demand by using the host-guest mechanism, because guest changing can induce much larger structural transformation and guest loading can be easily disturbed in the open environment. From the view point of framework/material design, it is more direct and predictable to tune the thermal expansion of the framework by changing the metal nodes and/or the organic linkers while maintaining the connection of the framework (Chapman et al., 2006; Korcok et al., 2009). Actually, the variation of metal nodes often affects the flexibility and dynamic responsive behaviors of the framework materials (Millange et al., 2008; Choi et al., 2010; Wang et al., 2011; He et al., 2017), but it has been rarely utilized to regulate the thermal expansion behaviors of MOFs, since the structural variations in such systems are always very small and difficult to quantitatively visualized.

Herein, we report a series of isostructral fence-like MOFs [M(pba)_2_]·2DMA (**1**; MCF-52; M = Ni^2+^, Co^2+^, Zn^2+^, Cd^2+^, or mixed Zn^2+^/Cd^2+^; Hpba = (3-pyridin-4-yl)benzoic acid; DMA = *N*,*N*-dimethylacetamide) exhibiting huge and controllable PTE and NTE (α_*a*_ = 154~228 × 10^−6^ K^−1^, α_*b*_ = 41~164 × 10^−6^ K^−1^ and α_*c*_ = −37~−152 × 10^−6^ K^−1^) dependent on the metal-ion radius. The thermal expansion mechanisms were systematically studied by comparing their single-crystal structures at different temperatures.

## Materials and methods

### Materials and instruments

All commercially available reagents and solvents were used as received without further purification. The ligand Hpba was synthesized according to the reported procedure (Zhou et al., 2013). Elemental (C, H, N) analyses (EA) were performed with a Vario EL elemental analyzer. Infrared (IR) spectra were recorded with a Bruker TENSOR 27 Fourier transform FT-IR spectrophotometer on KBr pellets in the range of 4,000–400 cm^−1^. Inductively coupled plasma-atomic emission spectroscopy (ICP-AES) results were collected by an Optima8300 coupled plasma-atomic emission spectrophotometer. Powder X-ray diffraction (PXRD) patterns were collected on a Bruker D8 ADVANCE X-ray powder diffractometer (Cu Kα, λ = 1.5418 Å). Scanning electron microscopy (SEM) and energy dispersive spectroscopy (EDS) images were recorded on a Quanta 400 field-emitted SEM device. Thermogravimetry (TG) analyses were performed on a TA Q50 instrument with a ramp rate of 10°C min^−1^ under a nitrogen flow. The high-pressure CO_2_ adsorption isotherm was measured on an automatic volumetric BELSORP-HP sorption apparatus in the range of 0–40 bar at 298 K. Prior to the sorption measurement, the as-synthesized sample was placed in the sample tube and dried under high vacuum at 230°C for 6 h to remove the solvent guests.

### Synthesis

Hpba (0.80 g, 4.0 mmol) was dissolved in DMA (60 mL) using a 250-mL vial, to which a DMA solution of Cd(NO_3_)_2_·6H_2_O (0.1 mol/L, 20 mL) and methanol (40 mL) were sequentially added. The mixture was then sealed with a screw cap, and heated to 90°C for 72 h. Colorless block-like crystals of [Cd(pba)_2_]·2DMA (MCF-52-Cd) were filtered and washed by DMA (yielded 1090 mg, ca. 80%). EA calcd (%) for [Cd(pba)_2_]·2DMA (C_32_H_34_N_4_O_6_Cd): C 56.27, H 5.02, N 8.20; Found: C 56.04, H 4.97, N 8.37. IR: 432(m), 472(w), 548(s), 589(m), 626(s), 682(s), 694(m), 769(s), 825(w), 845(m), 924(w), 1016(m), 1064(w), 1185(w), 1223(w), 1270(w), 1296(w), 1391(s), 1436(m), 1504(w), 1542(s), 1612(s),1637(m), 2938(w), 3071(w).

### Crystallography

Single-crystal X-ray diffraction (SCXRD) data were recorded on an Agilent SuperNova CCD diffractometer using mirror-monochromated Cu Kα radiation. The single crystals of [M(pba)_2_]·2DMA were mounted on the top of a glass fiber. The test temperature was controlled by dry N_2_ open flow using a Cryostream Plus cooler system, and corrected by a thermal couple at the crystal position. The variable-temperature unit-cell parameters (Tables S1–S5) were obtained by indexing the diffraction spots obtained with 30 diffraction images. Absorption corrections were applied by using the multi-scan program *CrysAlisPro*. The crystal structures were solved through the direct method and developed by the difference Fourier technique using the *SHELXTL* software package. Anisotropic thermal parameters were used to refine all non-hydrogen atoms of the frameworks. Hydrogen atoms were generated geometrically and the positions were refined in the riding mode. Crystallographic data are provided in Datasheet 1 and structural refinement details are summarized in Tables S6–S10. CCDC 1844211–1844220 for [M(pba)_2_]·2DMA contain the supplementary crystallographic data.

## Results and discussion

### Synthesis, structure, stability, and porosity

**Figure 1:**
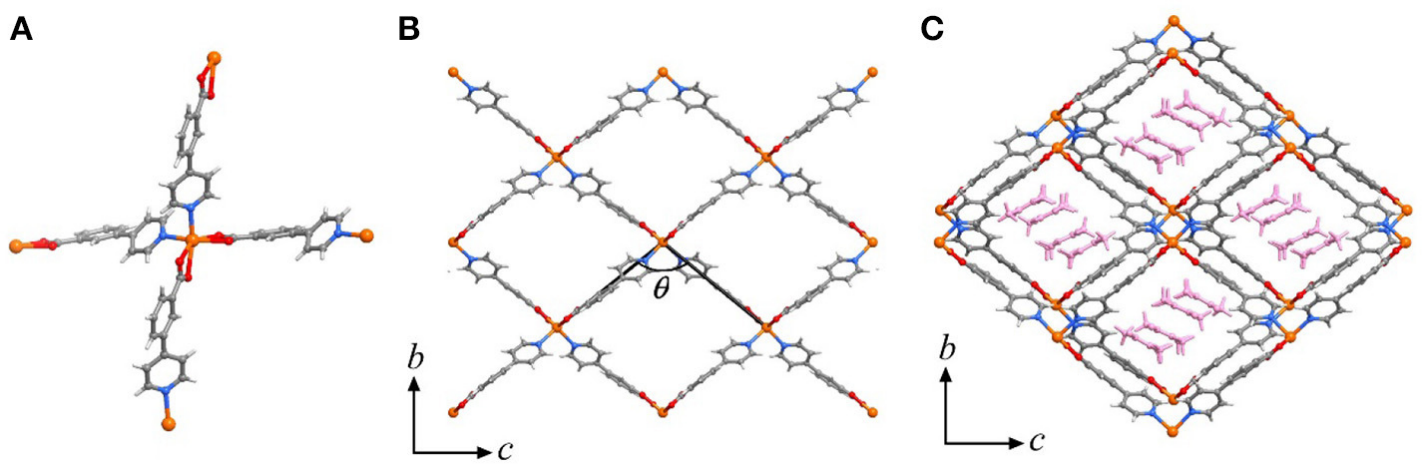
Perspective views of the crystal structure of [M(pba)_2_]·2DMA. **(A)** Coordination environment of the metal node. **(B)** The metal-organic fence, and the definition of its interior angle. **(C)** The stacking structure of metal-organic fences with DMA as guests, which are highlighted in pink. Metal ion: orange, C: dark gray, H: light gray, N: blue, O: red.

**Figure 2:**
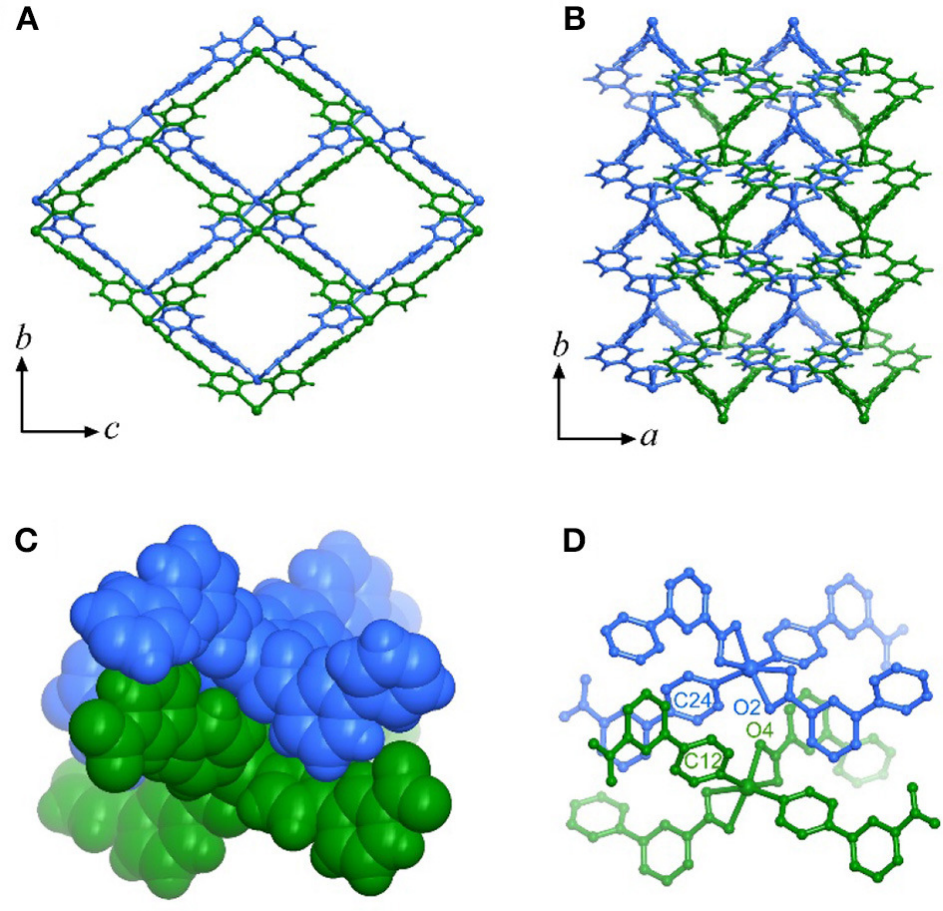
The stacking mode of the metal-organic fences. **(A)** Top view of the stacking layers. **(B)** Side view of the stacking layers. **(C)** Stacking of the tetrahedral building units of adjacent layers (highlighted in the space-filling mode). **(D)** The key atoms of adjacent tetrahedral building units.

High-quality single-crystal samples of [Cd(pba)_2_]·2DMA can be obtained through solvothermal reaction of Cd(NO_3_)_2_ and Hpba in mixed solvent DMA/methanol at 90°C. SCXRD revealed that [Cd(pba)_2_]·2DMA crystallizes in the orthogonal space group *P*2_1_2_1_2_1_, containing one Cd^2+^ ion with pseudo-octahedron coordination configuration, two bent pba^−^ ligands and two DMA guest molecules in its asymmetric unit. Each Cd^2+^ ion is coordinated by two carboxylate groups and two pyridyl groups from four pba^−^ ligands with a tetrahedral configuration, in which the carboxylate group exhibits the bidentate chelating mode. Each pba^−^ ligand coordinates with two Cd^2+^ ions by using its carboxylate and pyridyl ends. Considering Cd^2+^ ions as 4-connected nodes and pba^−^ ligands as linkers, the coordination network can be simplified as a two-dimensional (2D) rhombus grid or a typical hinged fence with the 4-connected **sql** topology parallel to the *bc*-plane (Figure 1). Such 2D grids stack in a zigzag-offset fashion via C–H···O hydrogen bonds and C–H···π interactions to form the 3D supramolecular structure (Figure 2). Thanks to the long ligands, after layers stacking, 1D rhombus nano-channels with a cross-sectional area up to 8.5 × 11.3 Å^2^ are formed along the *a*-axis direction and the solvent accessible void reaches 46%. The DMA guests are packed closely in a “face to face” way in the channels (Figure 1C).

Comparison between the measured PXRD pattern and the simulated one shows the high purity of the [Cd(pba)_2_]·2DMA samples (Figure S1). Then we also studied the stability and porosity of [Cd(pba)_2_]·2DMA. The TG curve (Figure S2) showed no weight loss below 80°C. Although the pore diameter is large, the relatively high boiling point and closely packing of DMA guest can effectively limit the guest escape. Then a steady plateau starts up from 230°C until decomposition above 350°C, and the weight loss of 26.4%, according with the theoretical value of 25.6%, meaning that the guests can be removed completely. Thus, the as-synthesized samples of [Cd(pba)_2_]·2DMA were activated by heating at 230°C under vacuum. The PXRD pattern shows the crystallinity can't maintain well after activation, which may be attributed to that the entire framework is formed by stacking 2D layers through the relatively weak van der Waals force. Interestingly, activated [Cd(pba)_2_] can revert to its original crystallinity after exposed to the DMA vapor. In addition, high pressure sorption experiment showed that activated [Cd(pba)_2_] can adsorb considerable amount of CO_2_ at 298 K and 40 bar (Figure S3), giving a pore volume of 0.16 cm^3^/g and a BET surface area of 347 m^2^/g. These results indicated that the desolvation-induced amorphism arises from local distortions of the coordination framework rather than destroying the framework connections.

### Thermal expansion properties

**Figure 3:**
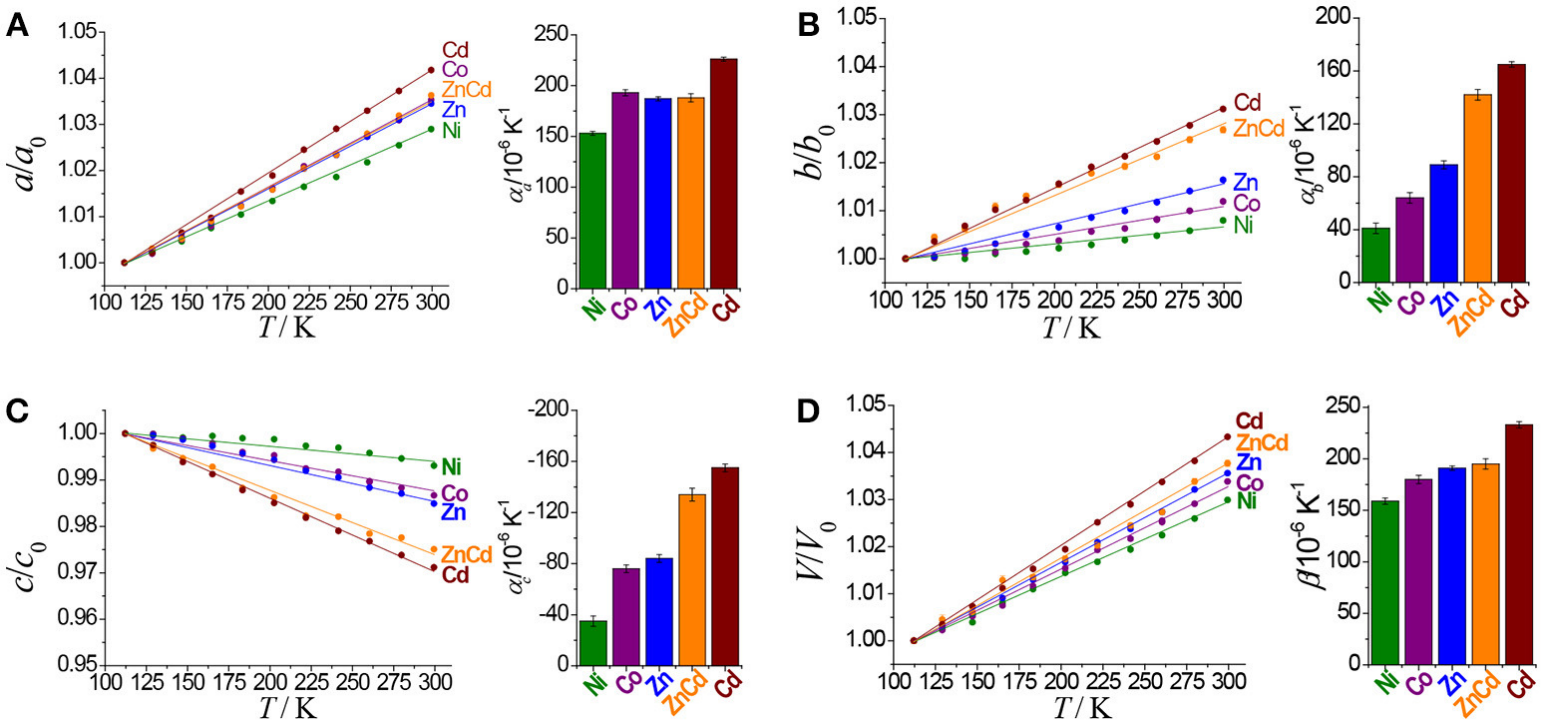
Thermal expansion behaviors of [M(pba)_2_]·2DMA.

The hinged-fence structural models may bring notable thermal expansion behaviors (DeVries et al., 2011; Zhou et al., 2013, 2015). In situ variable-temperature SCXRD measurements in the range of 112–300 K were used to characterize the thermal expansion properties of [Cd(pba)_2_]·2DMA (Figure 3 and Table 1). Encouragingly, [Cd(pba)_2_]·2DMA possesses the exceptionally large thermal expansion coefficients. From 112 to 300 K, the *a*-, *b*-, and *c*-axes of [Cd(pba)_2_]·2DMA change +4.2, +3.1, and −2.9%, respectively, giving linear thermal expansion coefficients α_*a*_ = +226 × 10^−6^ K^−1^, α_*b*_ = +165 × 10^−6^ ${\text{K}}_{,}^{-1}$ and α_*c*_ = −155 × 10^−6^ K^−1^. As a combined result, the volume increased by 4.3%, corresponding to a thermal expansion coefficient of +233 × 10^−6^ K^−1^. It is worth noting that this is the first solid material exhibiting such huge thermal expansion with all axial thermal expansion coefficients |α| > 150 × 10^−6^ K^−1^ (Goodwin et al., 2008; Das et al., 2010; Zhou et al., 2015).

**Table 1:** Thermal expansion coefficients of this series of isostructral metal-organic fences based on different metal ions.

	**r_ion_ / Å**	***α_*a*_*/10^−6^ K^−1^**	***α_*b*_*/10^−6^ K^−1^**	***α_*c*_*/10^−6^ K^−1^**	***β_*V*_*/10^−6^ K^−1^**
[Ni(pba)_2_]·2DMA	0.69	153(2)	41(4)	−35(4)	159(3)
[Co(pba)_2_]·2DMA	0.72	193(3)	64(4)	−76(3)	180(4)
[Zn(pba)_2_]·2DMA	0.74	187(2)	89(3)	−84(3)	191(2)
[Zn_0.77_Cd_0.23_(pba)_2_]· 2DMA	0.74~0.96	188(4)	142(4)	−134(5)	195(5)
[Cd(pba)_2_]·2DMA	0.96	226(2)	165(2)	−155(3)	233(3)

To clarify the structural origin of such huge expansion behaviors of these isostructural compounds, their single-crystal structures were determined at 112 K and 300 K. Structural analyses revealed that the interlayer interactions are mainly C–H···π interactions between the aromatic rings. From 112 to 300 K, the two shortest C–H···π separations, C6–H6···C22 and C18–H18···C10, increased from 3.036(5) and 3.115(5) Å to 3.128(5) and 3.285(5) Å, respectively, resulting in an increase of the interlayer distance from 5.750(1) to 5.978(1) Å and the huge PTE of the *a*-axis. The temperature change also causes the hinged-fence effect of the 2D rhombus grids parallel to the *bc*-plane, resulting in the reverse thermal expansion behavior of the *b*- and *c*-axes. The angle between adjacent pyridyl groups of the grid is defined as θ (Figure 1B). As the temperature rises from 112 to 300 K, θ decreases from 101.8° to 98.5°, causing the deformation of the entire metal-organic fence with the PTE of the *b*-axis and the NTE of the *c*-axis (Table 2). The comparison of structural detail between 112 and 300 K shows that the changes are very small in coordination bond lengths (Δ_max_ < 0.02 Å, Table S11) but very large in the coordination bond angles (Δ_max_ > 4°, Tables S12–S16). In other words, the distortion of the metallic coordination octahedron is the main source of such a huge thermal expansion.

**Table 2:** Temperature induced variation of the interior angle θ (°) for the metal-organic fences.

	**[Ni(pba)_2_]· 2DMA**	**[Co(pba)_2_]· 2DMA**	**[Zn(pba)_2_]· 2DMA**	**[Zn_0.77_Cd_0.23_ (pba)_2_]·2DMA**	**[Cd(pba)_2_]· 2DMA**
112 K	95.271(3)	96.625(5)	96.342(3)	98.809(4)	101.844(4)
300 K	94.428(3)	95.223(4)	94.602(3)	95.912(3)	98.499(4)
Δ	−0.843	−1.402	−1.740	−2.897	−3.345

Replacing Cd(NO_3_)_2_·6H_2_O by Ni(NO_3_)_2_·6H_2_O, Co(NO_3_)_2_·6H_2_O, or Zn(NO_3_)_2_·6H_2_O in the synthesis gave isostructural crystals of [Ni(pba)_2_]·2DMA, [Co(pba)_2_]·2DMA, and [Zn(pba)_2_]·2DMA, respectively (Figure S4). SCXRD from 112 to 300 K showed that the thermal expansion magnitude of these compounds is in the same order of their metal ion radii, i.e., Ni (0.69 Å) < Co (0.72 Å) < Zn (0.74 Å) < Cd (0.92 Å). Specifically, as the radii of the metal ions increases from 0.69 Å to 0.92 Å, the thermal expansion coefficient of the *a*-axis increases by 48%, while the thermal expansion coefficients of the *b*- and *c*-axes increase by more than 300% (Figure 3 and Table 1). In other words, the metal ion has a significant impact for the thermal expansion of the *bc*-plane showing the hinged-fence effect. Analyzing the crystal-structure details showed that, from 112 to 300 K, the maximal variation of coordination bond angles is less than 1.5° in [Ni(pba)_2_]·2DMA but more than 4.0° in [Cd(pba)_2_]·2DMA (Tables S12–S16), meanwhile the fence angle θ decreases by only 0.8° in [Ni(pba)_2_]·2DMA but decreases by 3.3° in [Cd(pba)_2_]·2DMA (Table 2). Thus, it can be seen that the increase of metal ion radius can effectively enlarge the distortion of the coordination octahedron and enhance the hinged-fence effect.

**Figure 4:**
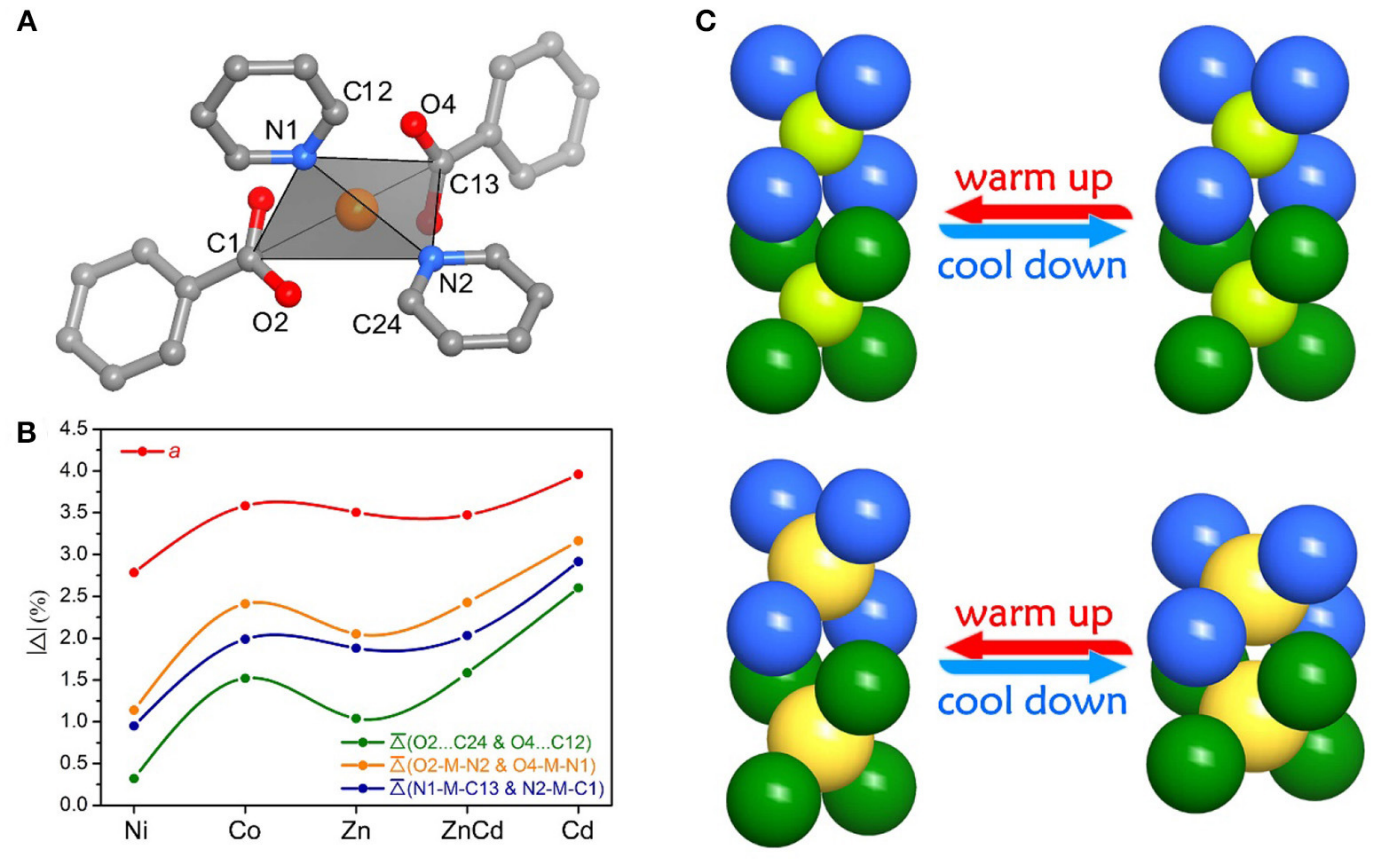
Elucidation of the thermal expansion mechanism for the fence stacking. **(A)** The tetrahedral building unit. **(B)** The selected parameters and variations for the geometry of the tetrahedral building unit and the interlayer separation/interaction. **(C)** Schematic views of thermal expansion upon the change of steric hindrance and metal ions.

The relationship between the PTE of the *a*-axis and the metal ion radius can be explained by the change of steric hindrance. Each metal ion coordinates with two carboxyl groups and two pyridyl groups arranged in a tetrahedral configuration. The tetrahedral building units in adjacent layers intercalate into each other. The shapes of the tetrahedral units, defined by the locations of the pyridyl groups and carboxyl groups, control the steric hindrance between the interlayer tetrahedra. Detailed analyses of crystal structures with different metal ions showed that the larger metal ions correspond to the more open tetrahedra (O2···C24 and O4···C12 between the pyridyl groups and carboxyl groups), smaller steric hindrance, and shorter interlayer separation (i.e., the *a*-axis length). Further, the larger the metal ions allow the coordination tetrahedra to be more flexible, leading to the greater changes of the interlayer separations and the larger PTE of the *a*-axis (Figure 4 and Table S17).

**Figure 5:**
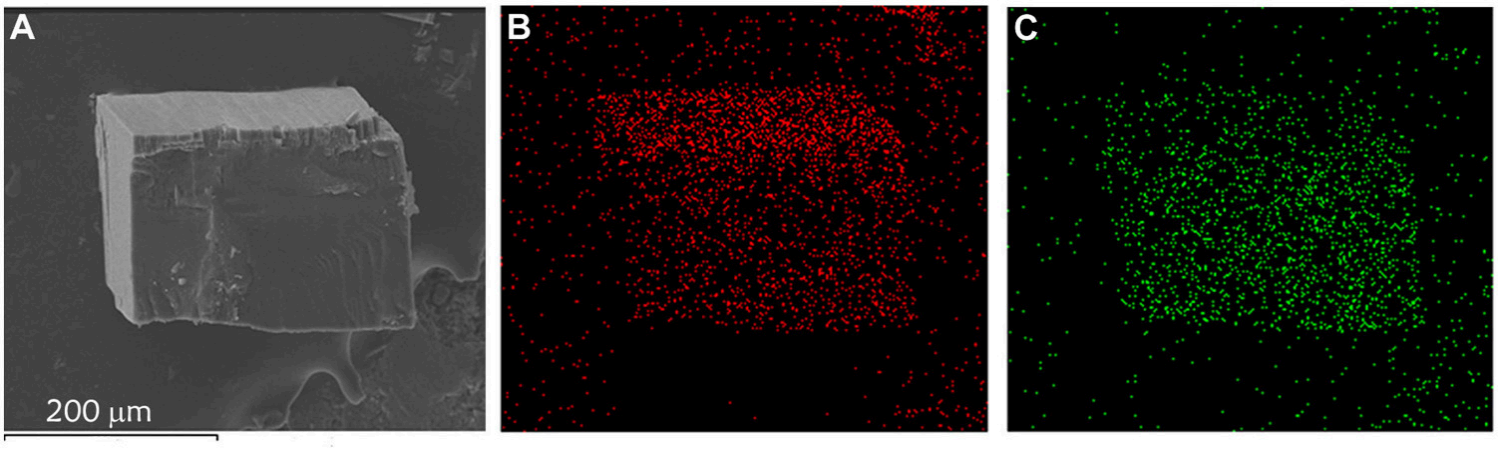
SEM and EDS images of [Zn_0.77_Cd_0.23_(pba)_2_]·2DMA. **(A)** SEM image of [Zn_0.77_Cd_0.23_(pba)_2_]·2DMA. **(B)** EDS image of zinc. **(C)** EDS image of cadmium.

Solid-solution-type MOFs have been successfully used to achieve adsorption properties between those of two corresponding parent phases (Deng et al., 2010; Horike et al., 2012; Chen et al., 2015; Inukai et al., 2015), but this strategy has not been applied to control thermal expansion behaviors. Preliminary trial showed that using a 1:1 mixture of Cd(NO_3_)_2_·6H_2_O and Zn(NO_3_)_2_·6H_2_O as starting material can give solid-solution crystals [Zn_0.77_Cd_0.23_(pba)_2_]·2DMA, in which the Zn/Cd ratio was determined by ICP-AES measurement of the acid-digested samples. SEM and EDS images revealed that Zn^2+^ and Cd^2+^ ions were uniformly distributed in the crystal (Figure 5). Variable-temperature SCXRD showed that the unit-cell parameters of [Zn_0.77_Cd_0.23_(pba)_2_]·2DMA are in between those of [Zn(pba)_2_]·2DMA and [Cd(pba)_2_]·2DMA (Figure 3 and Table 1). Free refinements of the single-crystal structures of [Zn_0.77_Cd_0.23_(pba)_2_]·2DMA at 112 and 300 K gave the Zn^2+^/Cd^2+^ occupancy of 0.75/0.25 (Table S10), in agreement with the results of ICP-AES. As expected, the thermal expansion magnitude of its *bc*-plane is between that of [Zn(pba)_2_]·2DMA and [Cd(pba)_2_]·2DMA. The structural details show that, from 112 to 300 K, the temperature-induced variation of the fence angle θ is 2.9° between 1.7° of [Zn(pba)_2_]·2DMA and 3.3° of [Cd(pba)_2_]·2DMA (Table 2). Unexpectedly, the thermal expansion of the *a*-axis of [Zn_0.77_Cd_0.23_(pba)_2_]·2DMA approximates to that of [Zn(pba)_2_]·2DMA, rather than in between those of [Zn(pba)_2_]·2DMA and [Cd(pba)_2_]·2DMA. As mentioned above, the thermal expansion behavior of the *a*-axis depends on the distance between the vertices of the tetrahedral units and the corresponding steric hindrance. In [Zn_0.77_Cd_0.23_(pba)_2_]·2DMA, the smaller ion Zn(II) with the larger steric hindrance may restrain the proximity of the adjacent layers, so that its thermal expansion behavior of *a*-axis is mainly dependent on Zn(II).

## Conclusions

In summary, a series of isostructral metal-organic fences were obtained using the bent organic ligand with Ni(II), Co(II), Zn(II), and Cd(II). Due to the van der Waals interactions between these stacking 2D metal organic layers, the interlayer distance is particularly sensitive to temperature change, resulting in extraordinarily large PTE. On the other hand, the hinged-fence effect occurs across the metal-organic fence, causing cooperative large PTE and NTE behaviors. Interestingly, the larger ions possessing more flexible coordination geometries can not only induce larger fencing actions but also control the inter-layer steric hindrance, leading to larger thermal expansion magnitudes. Overall, this series of compounds exhibit abundant thermal expansion mechanisms and represent the first solid material with all axial thermal expansion coefficients |α| > 150 × 10^−6^ K^−1^.

## Author contributions

J-PZ planned the research and supervised the project. H-LZ carried out the syntheses, characterization, and crystal structure determination. H-LZ, J-PZ, and X-MC analyzed the data and co-wrote the manuscript.

### Conflict of interest statement

The authors declare that the research was conducted in the absence of any commercial or financial relationships that could be construed as a potential conflict of interest.
