# Supplementary material for: Controlling Thermal Expansion Behaviors of Fence-Like Metal-Organic Frameworks by Varying/Mixing Metal Ions
Source: Front Chem. 2018 Jul 24;6:306. doi: 10.3389/fchem.2018.00306 (PMC6066979; doi:10.3389/fchem.2018.00306)
Supplement: Supplementary file 1 [file Presentation_1.PDF]

Supplementary Material

Controlling Thermal Expansion Behaviors of Fence-like Metal-Organic Frameworks by Varying/Mixing Metal ions

Hao-Long Zhou[1], Jie-Peng Zhang[1]*, Xiao-Ming Chen[1,2]

[1]MOE Key Laboratory of Bioinorganic and Synthetic Chemistry, School of Chemistry, Sun Yat-Sen University, Guangzhou 510275, China

[2]Department of Chemistry and Key Laboratory for Preparation and Application of Ordered Structural Materials of Guangdong Province, Shantou University, Guangdong 515063, China

*** Correspondence:**
Jie-Peng Zhang
zhangjp7@mail.sysu.edu.cn

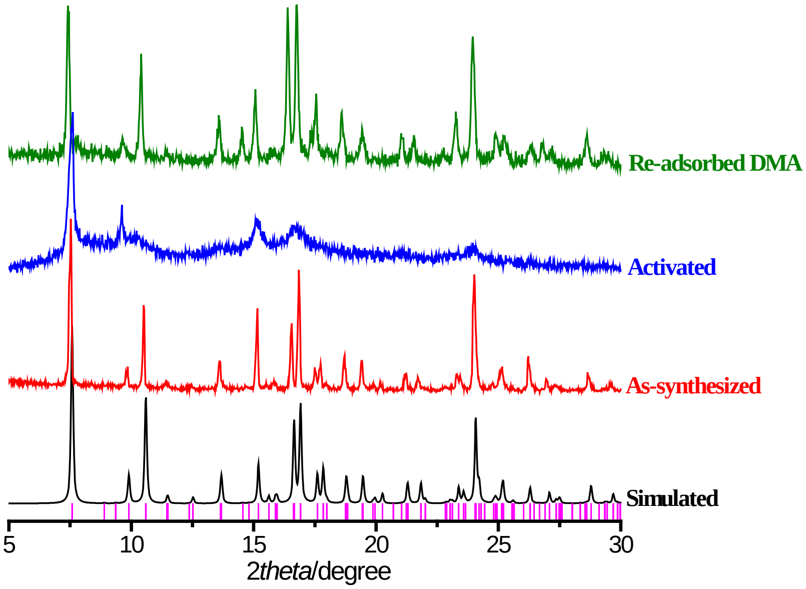

Figure S1 PXRD patterns for $[Cd(pba)_2]\cdot 2DMA$.

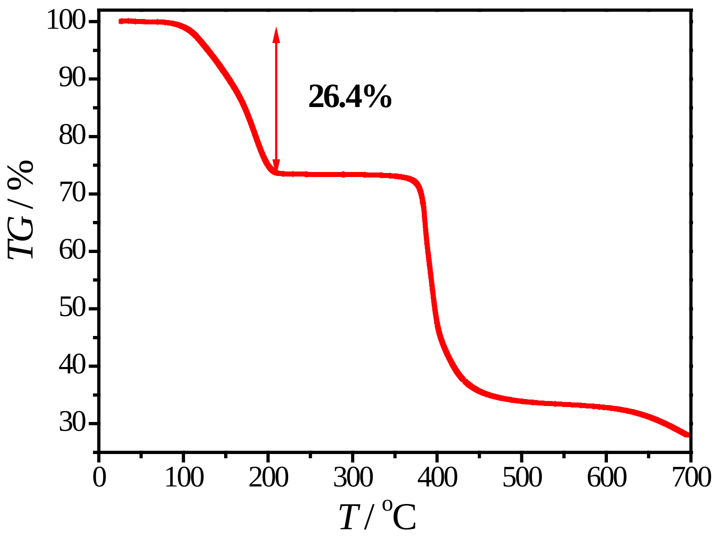

Figure S2 TG curve for [Cd(pba)$_2$]·2DMA.

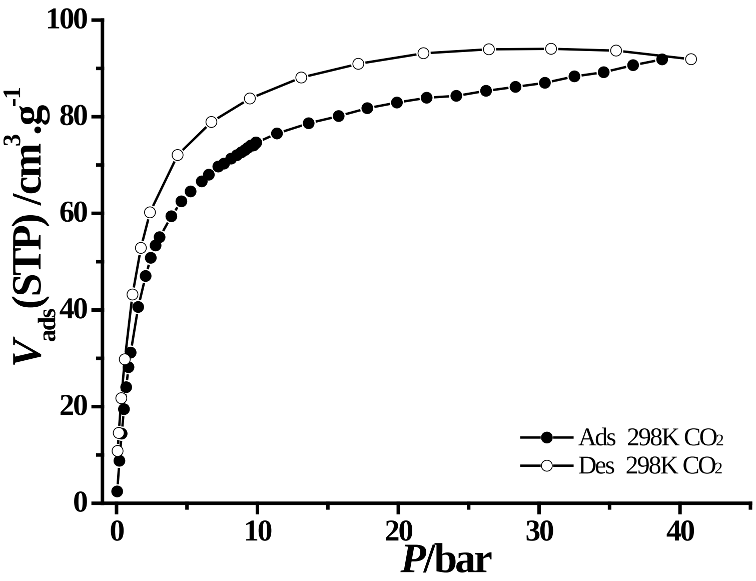

Figure S3 High-pressure CO_2 sorption isotherms measured at 298 K for [Cd(pba)$_2$]·2DMA.

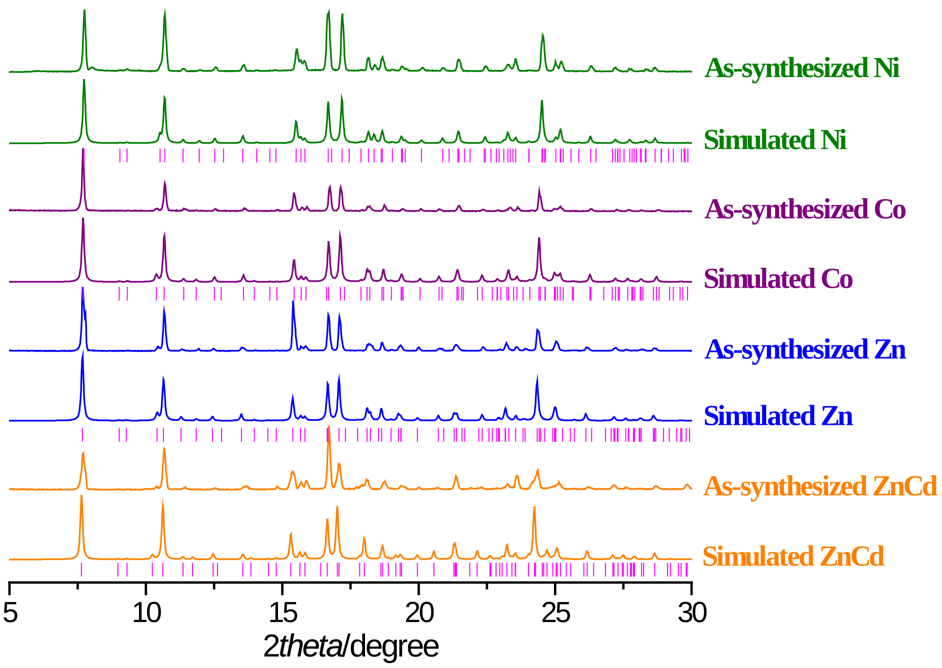

Figure S4 PXRD patterns for $[Ni(pba)_2]\cdot 2DMA$ (green), $[Co(pba)_2]\cdot 2DMA$ (purple), $[Zn(pba)_2]\cdot 2DMA$ (blue), and $[Zn_{0.77}Cd_{0.23}(pba)_2]\cdot 2DMA$ (orange).

Table S1 The variable-temperature unit-cell parameters of $[Ni(pba)_2]\cdot 2DMA$.

T/K	*a*/Å	*b*/Å	*c*/Å	*V*/Å^3
299(1)	12.003(3)	15.552(4)	16.797(3)	3135(1)
260(1)	11.919(2)	15.503(3)	16.843(2)	3112.2(9)
222(1)	11.857(2)	15.474(3)	16.869(2)	3095(1)
183(1)	11.787(2)	15.452(3)	16.897(2)	3077.4(9)
147(1)	11.719(3)	15.429(3)	16.899(3)	3056(1)
112(1)	11.665(3)	15.429(3)	16.914(3)	3044(1)
129(1)	11.693(2)	15.431(3)	16.912(3)	3051.4(9)
165(1)	11.753(2)	15.445(3)	16.905(2)	3068.7(9)
203(1)	11.821(3)	15.463(4)	16.893(3)	3088(1)
241(1)	11.882(3)	15.489(3)	16.862(2)	3103(1)
280(1)	11.962(4)	15.519(4)	16.823(3)	3123(1)

Table S2 The variable-temperature unit-cell parameters of $[Co(pba)_2]\cdot 2DMA$.

T/K	*a*/Å	*b*/Å	*c*/Å	*V*/Å^3
299(1)	11.964(2)	15.524(2)	17.028(3)	3162.5(9)
260(1)	11.872(2)	15.467(2)	17.081(4)	3137(1)
222(1)	11.798(2)	15.428(2)	17.128(4)	3118(1)
183(1)	11.699(2)	15.3877(19)	17.189(4)	3095(1)
147(1)	11.614(3)	15.3575(19)	17.239(4)	3075(1)
112(1)	11.556(4)	15.341(2)	17.258(5)	3059(1)
129(1)	11.579(3)	15.346(2)	17.256(4)	3066(1)
165(1)	11.648(3)	15.363(2)	17.222(4)	3082(1)
203(1)	11.743(2)	15.3990(17)	17.177(3)	3106.1(9)
241(1)	11.828(2)	15.438(2)	17.116(4)	3125(1)
280(1)	11.913(2)	15.4939(19)	17.056(4)	3148(1)

Table S3 The variable-temperature unit-cell parameters of $[Zn(pba)_2]\cdot 2DMA$.

T/K	*a*/Å	*b*/Å	*c*/Å	*V*/Å^3
299(1)	11.9740(14)	15.6689(13)	16.9813(15)	3186.0(5)
260(1)	11.8911(15)	15.5974(15)	17.0407(16)	3160.6(6)
222(1)	11.8106(15)	15.5485(14)	17.1033(15)	3140.8(6)
183(1)	11.7170(12)	15.4934(12)	17.1665(13)	3116.4(5)
147(1)	11.6383(14)	15.4416(14)	17.2186(15)	3094.4(5)
112(1)	11.5746(14)	15.4159(15)	17.2417(14)	3076.5(5)
129(1)	11.6085(14)	15.4241(14)	17.2346(13)	3085.8(5)
165(1)	11.6752(15)	15.464(15)	17.1948(15)	3104.4(6)
203(1)	11.7584(15)	15.5172(15)	17.1439(15)	3128.0(6)
241(1)	11.8448(16)	15.5691(14)	17.0791(16)	3149.6(6)
280(1)	11.9343(13)	15.6333(13)	17.0189(15)	3175.3(5)

Table S4 The variable-temperature unit-cell parameters of $[Zn_{0.77}Cd_{0.23}(pba)_2]\cdot 2DMA$.

T/K	*a*/Å	*b*/Å	*c*/Å	*V*/Å^3
299(1)	11.9839(18)	15.575(10)	17.262(5)	3222(2)
260(1)	11.8874(15)	15.490(6)	17.321(4)	3190(2)
222(1)	11.8006(17)	15.438(7)	17.390(4)	3168(2)
183(1)	11.7053(16)	15.366(8)	17.495(5)	3147(2)
147(1)	11.624(2)	15.262(10)	17.608(7)	3124(2)
112(1)	11.564(2)	15.168(8)	17.703(6)	3105(2)
129(1)	11.600(3)	15.236(10)	17.646(8)	3119(2)
165(1)	11.668(2)	15.333(12)	17.576(6)	3145(2)
203(1)	11.7473(17)	15.402(9)	17.460(5)	3159(2)
241(1)	11.8349(19)	15.460(10)	17.386(6)	3181(2)
280(1)	11.933(2)	15.544(10)	17.306(5)	3210(2)

Table S5 The variable-temperature unit-cell parameters of $[Cd(pba)_2]\cdot 2DMA$.

T/K	*a*/Å	*b*/Å	*c*/Å	*V*/Å^3
299(1)	11.947(4)	15.381(3)	17.835(2)	3277(1)
260(1)	11.846(4)	15.280(3)	17.938(2)	3247(1)
222(1)	11.749(4)	15.201(3)	18.031(2)	3220(1)
183(1)	11.645(4)	15.098(3)	18.14(2)	3189(1)
147(1)	11.543(4)	15.018(2)	18.251(2)	3164(1)
112(1)	11.468(3)	14.916(2)	18.3640(18)	3141(1)
129(1)	11.493(4)	14.970(2)	18.3184(19)	3152(1)
165(1)	11.580(4)	15.068(2)	18.203(2)	3176(1)
203(1)	11.685(4)	15.149(3)	18.089(2)	3202(1)
241(1)	11.801(4)	15.234(3)	17.979(2)	3232(1)
280(1)	11.895(4)	15.330(3)	17.883(2)	3261(1)

Table S6 Crystal data and structural refinement results for $[Ni(pba)_2]\cdot 2DMA$.

Complex	$[Ni(pba)_2]\cdot 2DMA$	
Formula	$C_{32}H_{34}NiN_4O_6$	$C_{32}H_{34}NiN_4O_6$
Formula weight	629.34	629.34
Temperature/K	112(2)	300(2)
Crystal system	Orthorhombic	Orthorhombic
Space group	$P2_12_12_1$	$P2_12_12_1$
a/Å	11.6694(2)	11.9944(2)
b/Å	15.4264(3)	15.5593(3)
c/Å	16.9159(3)	16.8124(2)
V/Å^3	3045.15(10)	3137.61(9)
Z	4	4
D_c /g cm^{-3}	1.373	1.332
reflns coll.	7896	8304
unique reflns	5438	5564
R_{int}	0.0165	0.0167
R_1 $[I > 2\sigma(I)]$[a]	0.0288	0.0350
wR_2 $[I > 2\sigma(I)]$[b]	0.0762	0.0933
R_1 (all data)	0.0356	0.0415
wR_2 (all data)	0.0821	0.1008
GOF	1.089	1.042
Flack	0.01	0.03

Table S7 Crystal data and structural refinement results for $[Co(pba)_2]\cdot 2DMA$.

Complex	$[Co(pba)_2]\cdot 2DMA$	
Formula	$C_{32}H_{34}CoN_4O_6$	$C_{32}H_{34}CoN_4O_6$
Formula weight	629.56	629.56
Temperature/K	112(2)	300(2)
Crystal system	Orthorhombic	Orthorhombic
Space group	$P2_12_12_1$	$P2_12_12_1$
a/Å	11.5495(2)	11.9634(2)
b/Å	15.3560(3)	15.5365(2)
c/Å	17.2435(3)	17.0235(2)
V/Å^3	3058.21(10)	3164.15(8)
Z	4	4
D_c /g cm^{-3}	1.367	1.322
reflns coll.	6884	7275
unique reflns	4835	5235
R_{int}	0.0359	0.0301
R_1 [$I > 2\sigma(I)$][a]	0.0396	0.0418
wR_2 [$I > 2\sigma(I)$][b]	0.0943	0.1049
R_1 (all data)	0.0561	0.0565
wR_2 (all data)	0.1046	0.1170
GOF	0.976	1.045
Flack	-0.02	-0.01

Table S8 Crystal data and structural refinement results for $[Zn(pba)_2]\cdot 2DMA$.

Complex	$[Zn(pba)_2]\cdot 2DMA$	
Formula	$C_{32}H_{34}ZnN_4O_6$	$C_{32}H_{34}ZnN_4O_6$
Formula weight	636.00	636.00
Temperature/K	112(2)	300(2)
Crystal system	Orthorhombic	Orthorhombic
Space group	$P2_12_12_1$	$P2_12_12_1$
a/Å	11.5766(2)	11.98240(11)
b/Å	15.4336(3)	15.65966(16)
c/Å	17.2458(3)	16.97628(14)
V/Å^3	3081.28(10)	3185.43(5)
Z	4	4
D_c /g cm^{-3}	1.371	1.326
reflns coll.	7574	7655
unique reflns	5105	5407
R_{int}	0.0109	0.0124
R_1 [$I > 2\sigma(I)$][a]	0.0273	0.0306
wR_2 [$I > 2\sigma(I)$][b]	0.0780	0.0851
R_1 (all data)	0.0301	0.0329
wR_2 (all data)	0.0810	0.0878
GOF	1.089	1.042
Flack	0.04	0.03

Table S9 Crystal data and structural refinement results for $[Cd(pba)_2]\cdot 2DMA$.

Complex	$[Cd(pba)_2]\cdot 2DMA$	
Formula	$C_{32}H_{34}CdN_4O_6$	$C_{32}H_{34}CdN_4O_6$
Formula weight	683.03	683.03
Temperature/K	112(2)	300(2)
Crystal system	Orthorhombic	Orthorhombic
Space group	$P2_12_12_1$	$P2_12_12_1$
a/Å	11.5002(3)	11.9555(1)
b/Å	14.9226(3)	15.3787(2)
c/Å	18.3781(4)	17.8504(2)
V/Å^3	3153.92(12)	3281.98(6)
Z	4	4
D_c /g cm^{-3}	1.438	1.382
reflns coll.	7211	7480
unique reflns	5057	5552
R_{int}	0.0233	0.0260
R_1 $[I > 2\sigma(I)]$[a]	0.0324	0.0405
wR_2 $[I > 2\sigma(I)]$[b]	0.0920	0.1111
R_1 (all data)	0.0337	0.0427
wR_2 (all data)	0.0944	0.1164
GOF	1.077	1.049
Flack	0.02	-0.01

Table S10 Crystal data and structural refinement results for $[Zn_{0.77}Cd_{0.23}(pba)_2]\cdot 2DMA$.

Complex	$[Zn_{0.77}Cd_{0.23}(pba)_2]\cdot 2DMA$	$[Zn_{0.77}Cd_{0.23}(pba)_2]\cdot 2DMA$
Formula	$C_{32}H_{34}Zn_{0.75}Cd_{0.25}N_4O_6$	$C_{32}H_{34}Zn_{0.75}Cd_{0.25}N_4O_6$
Formula weight	647.76	647.76
Temperature/K	112(2)	300(2)
Crystal system	Orthorhombic	Orthorhombic
Space group	$P2_12_12_1$	$P2_12_12_1$
a/Å	11.5821(4)	11.9844(3)
b/Å	15.1741(5)	15.5676(4)
c/Å	17.7091(5)	17.2670(5)
V/Å^3	3112.34(17)	3221.47(15)
Z	4	4
D_c /g cm^{-3}	1.382	1.326
reflns coll.	6904	7036
unique reflns	5144	4805
R_{int}	0.0298	0.0246
R_1 [$I > 2\sigma(I)$][a]	0.0456	0.0388
wR_2 [$I > 2\sigma(I)$][b]	0.1211	0.1029
R_1 (all data)	0.0560	0.0449
wR_2 (all data)	0.1312	0.1116
GOF	1.033	1.034
Flack	-0.07	-0.04

Table S11 Coordination bond lengths (Å) for $[M(pba)_2]\cdot 2DMA$.

	112 K	300 K
Ni1-O1	2.1536(16)	2.1684(17)
Ni1-O2	2.0684(16)	2.0644(19)
Ni1-O3	2.1348(16)	2.1379(18)
Ni1-O4	2.0789(16)	2.0793(19)
Ni1-N2	2.0461(19)	2.057(2)
Ni1-N1	2.0564(19)	2.061(2)
Co1-O1	2.244(3)	2.244(2)
Co1-O2	2.073(2)	2.068(3)
Co1-O3	2.217(3)	2.201(3)
Co1-O4	2.079(2)	2.089(3)
Co1-N2	2.091(3)	2.099(3)
Co1-N1	2.092(3)	2.101(3)
Zn1-O1	2.3776(16)	2.4031(17)
Zn1-O2	2.0337(16)	2.0296(19)
Zn1-O3	2.2937(16)	2.2544(17)
Zn1-O4	2.0640(16)	2.0887(19)
Zn1-N2	2.074(2)	2.079(2)
Zn1-N1	2.086(2)	2.098(2)
Zn1/Cd1-O1	2.374(3)	2.373(3)
Zn1/Cd1-O2	2.115(4)	2.107(3)
Zn1/Cd1-O3	2.290(3)	2.268(3)
Zn1/Cd1-O4	2.139(5)	2.155(4)
Zn1/Cd1-N2	2.168(5)	2.172(3)
Zn1/Cd1-N1	2.173(4)	2.181(3)
Cd1-O1	2.392(3)	2.374(3)
Cd1-O2	2.291(3)	2.289(4)
Cd1-O3	2.349(3)	2.333(3)
Cd1-O4	2.312(3)	2.316(4)
Cd1-N2	2.284(3)	2.288(4)
Cd1-N1	2.293(3)	2.297(4)

Table S12 Coordination bond angles (°) for $[Ni(pba)_2]·2DMA$.

	112 K	300 K
O1-Ni1-O2	62.83(6)	62.57(8)
O1-Ni1-O3	89.13(6)	89.69(7)
O1-Ni1-O4	98.98(6)	99.47(8)
O1-Ni1-N2	160.27(7)	158.99(9)
O1-Ni1-N1	91.27(7)	91.43(8)
O2-Ni1-O3	99.71(6)	101.05(8)
O2-Ni1-O4	156.21(7)	157.01(8)
O2-Ni1-N2	97.67(7)	96.61(8)
O2-Ni1-N1	98.08(7)	98.24(8)
O3-Ni1-O4	62.92(6)	62.63(8)
O3-Ni1-N2	91.45(7)	91.91(8)
O3-Ni1-N1	160.20(7)	158.80(9)
O4-Ni1-N2	98.83(7)	99.86(8)
O4-Ni1-N1	97.50(7)	96.34(9)
N1-Ni1-N2	94.77(7)	94.55(9)

Table S13 Coordination bond angles (°) for $[Co(pba)_2]·2DMA$.

	112 K	300 K
O1-Co1-O2	61.33(9)	60.73(10)
O1-Co1-O3	90.09(9)	90.00(9)
O1-Co1-O4	95.71(10)	97.91(10)
O1-Co1-N2	160.67(11)	157.83(12)
O1-Co1-N1	89.69(11)	90.53(10)
O2-Co1-O3	96.36(10)	99.97(10)
O2-Co1-O4	149.44(11)	152.91(12)
O2-Co1-N2	99.51(12)	97.31(12)
O2-Co1-N1	100.46(12)	99.84(12)
O3-Co1-O4	61.62(9)	61.13(10)
O3-Co1-N2	89.62(11)	91.14(10)
O3-Co1-N1	160.69(12)	157.56(12)
O4-Co1-N2	101.12(12)	102.01(12)
O4-Co1-N1	99.19(12)	96.60(12)
N1-Co1-N2	96.86(12)	96.72(12)

Table S14 Coordination bond angles (°) for $[Zn(pba)_2]\cdot 2DMA$.

	112 K	300 K
O1-Zn1-O2	59.58(6)	58.88(7)
O1-Zn1-O3	87.34(5)	88.27(6)
O1-Zn1-O4	92.87(6)	94.04(7)
O1-Zn1-N2	160.41(7)	158.73(8)
O1-Zn1-N1	89.65(7)	89.57(7)
O2-Zn1-O3	95.02(6)	98.28(7)
O2-Zn1-O4	145.34(7)	147.42(8)
O2-Zn1-N2	101.22(7)	100.09(8)
O2-Zn1-N1	101.63(7)	101.48(8)
O3-Zn1-O4	60.60(6)	60.24(8)
O3-Zn1-N2	90.84(7)	92.11(7)
O3-Zn1-N1	158.65(7)	155.45(8)
O4-Zn1-N2	103.21(7)	104.60(8)
O4-Zn1-N1	98.50(7)	95.56(8)
N1-Zn1-N2	98.82(8)	98.59(8)

Table S15 Coordination bond angles (°) for $[Zn_{0.77}Cd_{0.23}(pba)_2]\cdot 2DMA$.

	112 K	300 K
O1-Zn1/Cd1-O2	58.78(16)	58.25(12)
O1-Zn1/Cd1-O3	91.45(11)	91.64(11)
O1-Zn1/Cd1-O4	95.19(15)	96.65(12)
O1-Zn1/Cd1-N2	159.36(17)	156.87(13)
O1-Zn1/Cd1-N1	88.98(13)	89.85(11)
O2-Zn1/Cd1-O3	97.80(16)	100.98(12)
O2-Zn1/Cd1-O4	146.67(17)	149.20(12)
O2-Zn1/Cd1-N2	100.64(17)	98.66(13)
O2-Zn1/Cd1-N1	101.06(16)	101.22(13)
O3-Zn1/Cd1-O4	59.58(16)	58.99(13)
O3-Zn1/Cd1-N2	89.89(16)	91.64(12)
O3-Zn1/Cd1-N1	158.17(17)	154.73(13)
O4-Zn1/Cd1-N2	103.21(17)	104.63(13)
O4-Zn1/Cd1-N1	98.64(17)	95.79(13)
N1-Zn1/Cd1-N2	97.31(17)	96.81(12)

Table S16 Coordination bond angles (°) for $[Cd(pba)_2]\cdot 2DMA$.

	112 K	300 K
O1-Cd1-O2	56.41(9)	55.65(13)
O1-Cd1-O3	96.77(9)	97.09(11)
O1-Cd1-O4	97.66(9)	99.61(13)
O1-Cd1-N2	157.43(10)	153.59(15)
O1-Cd1-N1	87.89(10)	89.09(13)
O2-Cd1-O3	99.67(9)	104.27(14)
O2-Cd1-O4	144.90(11)	148.60(16)
O2-Cd1-N2	101.10(11)	97.97(15)
O2-Cd1-N1	101.77(11)	101.19(15)
O3-Cd1-O4	56.76(9)	55.83(13)
O3-Cd1-N2	88.38(10)	90.16(13)
O3-Cd1-N1	156.84(10)	152.68(15)
O4-Cd1-N2	103.49(11)	105.41(15)
O4-Cd1-N1	100.17(11)	96.93(16)
N1-Cd1-N2	95.99(11)	96.04(15)

Table S17 The selected parameters (Å) and variations (%) of the geometry of the tetrahedral building unit and the interlayer separation/interaction.

T		[Ni(pba)$_2$]·2DMA	[Co(pba)$_2$]·2DMA	[Zn(pba)$_2$]·2DMA	[Zn$_{0.77}$Cd$_{0.23}$(pba)$_2$]·2DMA	[Cd(pba)$_2$]·2DMA
112 K	O2···C24	3.1962(28)	3.3191(50)	3.3425(29)	3.4881(64)	3.7143(43)
	O4···C12	3.2137(27)	3.3390(51)	3.3724(28)	3.4646(72)	3.7258(43)
	O2-M-N2	97.67(7)	99.51(12)	101.22(7)	100.64(17)	101.10(11)
	O4-M-N1	97.50(7)	99.19(12)	98.50(7)	98.64(17)	100.17(11)
	N1-M-C13	128.95(8)	130.11(13)	129.01(8)	128.42(17)	128.58(12)
	N2-M-C1	129.11(8)	130.41(13)	131.34(8)	130.25(18)	129.28(13)
	a	11.6694(2)	11.5495(2)	11.5766(2)	11.5821(4)	11.5002(3)
300 K	O2···C24	3.1851(33)	3.2783(52)	3.2873(33)	3.4379(58)	3.6112(65)
	O4···C12	3.2042(35)	3.2785(52)	3.3580(31)	3.4046(58)	3.6354(65)
	O2-M-N2	96.61(8)	97.31(12)	100.09(8)	98.66(13)	97.97(15)
	O4-M-N1	96.34(9)	96.60(12)	95.56(8)	95.79(13)	96.93(16)
	N1-M-C13	128.01(9)	127.36(13)	125.60(8)	125.28(14)	124.62(15)
	N2-M-C1	127.60(9)	127.98(13)	129.87(8)	128.14(14)	125.72(16)
	a	11.9944(2)	11.9634(2)	11.9824(1)	11.9844(3)	11.9555(1)
	Δ_1	-0.35	-1.23	-1.65	-1.44	-2.78
	Δ_2	-0.30	-1.81	-0.43	-1.73	-2.43
	Δ_3	-1.09	-2.21	-1.12	-1.97	-3.10
	Δ_4	-1.19	-2.61	-2.98	-2.89	-3.23
	Δ_5	-0.73	-2.11	-2.64	-2.45	-3.08
	Δ_6	-1.17	-1.86	-1.12	-1.62	-2.75
	Δ_a	2.79	3.58	3.51	3.47	3.96
