# Supplementary material for: Controlling Thermal Expansion Behaviors of Fence-Like Metal-Organic Frameworks by Varying/Mixing Metal Ions
Source: Front Chem. 2018 Jul 24;6:306. doi: 10.3389/fchem.2018.00306 (PMC6066979; doi:10.3389/fchem.2018.00306)

checkCIF/PLATON report

Structure factors have been supplied for datablock(s) Cd112K

THIS REPORT IS FOR GUIDANCE ONLY. IF USED AS PART OF A REVIEW PROCEDURE FOR PUBLICATION, IT SHOULD NOT REPLACE THE EXPERTISE OF AN EXPERIENCED CRYSTALLOGRAPHIC REFEREE.

No syntax errors found. CIF dictionary Interpreting this report

Datablock: Cd112K

Bond precision: C-C = 0.0056 A Wavelength=1.54178

Cell:	a=11.5002(3)	b=14.9226(3)	c=18.3781(4)
	alpha=90	beta=90	gamma=90
Temperature:	112 K		

	Calculated	Reported
Volume	3153.92(12)	3153.92(12)
Space group	P 21 21 21	P 21 21 21
Hall group	P 2ac 2ab	P 2ac 2ab
Moiety formula	C24 H16 Cd N2 O4, 2(C4 H9 N O)	C24 H16 Cd N2 O4, 2(C4 H9 N O)
Sum formula	C32 H34 Cd N4 O6	C32 H34 Cd N4 O6
Mr	683.04	683.03
Dx,g cm-3	1.439	1.438
Z	4	4
Mu (mm-1)	5.950	5.950
F000	1400.0	1400.0
F000’	1404.41	
h,k,lmax	14,18,22	13,18,22
Nref	6370[3571]	5057
Tmin,Tmax	0.350,0.304	0.485,1.000
Tmin’	0.265	

Correction method= # Reported T Limits: Tmin=0.485 Tmax=1.000
AbsCorr = MULTI-SCAN

Data completeness= 1.42/0.79 Theta(max)= 73.668

R(reflections)= 0.0324(4831) wR2(reflections)= 0.0944(5057)

S = 1.077 Npar= 394

The following ALERTS were generated. Each ALERT has the format
test-name_ALERT_alert-type_alert-level.
Click on the hyperlinks for more details of the test.

Alert level B

```
PLAT213_ALERT_2_B Atom O4              has ADP max/min Ratio .....        5.0 prolat
PLAT213_ALERT_2_B Atom C12             has ADP max/min Ratio .....        4.7 oblate
```

Alert level C

```
PLAT213_ALERT_2_C Atom O2              has ADP max/min Ratio .....        3.8 prolat
PLAT213_ALERT_2_C Atom C21             has ADP max/min Ratio .....        3.3 oblate
PLAT911_ALERT_3_C Missing # FCF Refl Between THmin & STh/L=  0.600         31 Report
PLAT915_ALERT_3_C No Flack x Check Done: Low Friedel Pair Coverage         58 %
PLAT972_ALERT_2_C Check Calcd Residual Density  0.78A From     Cd1      -2.28 eA-3
PLAT972_ALERT_2_C Check Calcd Residual Density  0.85A From     Cd1      -2.18 eA-3
PLAT972_ALERT_2_C Check Calcd Residual Density  0.72A From     Cd1      -1.53 eA-3
PLAT978_ALERT_2_C Number C-C Bonds with Positive Residual Density.          0 Note
```

Alert level G

```
PLAT004_ALERT_5_G Polymeric Structure Found with Maximum Dimension          2 Info
PLAT012_ALERT_1_G No     _shelx_res_checksum found in CIF ........     Please Check
PLAT380_ALERT_4_G Incorrectly? Oriented X(sp2)-Methyl Moiety .....        C30 Check
PLAT912_ALERT_4_G Missing # of FCF Reflections Above STh/L=  0.600         84 Note
PLAT913_ALERT_3_G Missing # of Very Strong Reflections in FCF ....          1 Note
```

---

0 **ALERT level A** = Most likely a serious problem - resolve or explain
2 **ALERT level B** = A potentially serious problem, consider carefully
8 **ALERT level C** = Check. Ensure it is not caused by an omission or oversight
5 **ALERT level G** = General information/check it is not something unexpected

1 ALERT type 1 CIF construction/syntax error, inconsistent or missing data
8 ALERT type 2 Indicator that the structure model may be wrong or deficient
3 ALERT type 3 Indicator that the structure quality may be low
2 ALERT type 4 Improvement, methodology, query or suggestion
1 ALERT type 5 Informative message, check

It is advisable to attempt to resolve as many as possible of the alerts in all categories. Often the minor alerts point to easily fixed oversights, errors and omissions in your CIF or refinement strategy, so attention to these fine details can be worthwhile. In order to resolve some of the more serious problems it may be necessary to carry out additional measurements or structure refinements. However, the purpose of your study may justify the reported deviations and the more serious of these should normally be commented upon in the discussion or experimental section of a paper or in the "special_details" fields of the CIF. checkCIF was carefully designed to identify outliers and unusual parameters, but every test has its limitations and alerts that are not important in a particular case may appear. Conversely, the absence of alerts does not guarantee there are no aspects of the results needing attention. It is up to the individual to critically assess their own results and, if necessary, seek expert advice.

Publication of your CIF in IUCr journals

A basic structural check has been run on your CIF. These basic checks will be run on all CIFs submitted for publication in IUCr journals (*Acta Crystallographica*, *Journal of Applied Crystallography*, *Journal of Synchrotron Radiation*); however, if you intend to submit to *Acta Crystallographica Section C* or *E* or *IUCrData*, you should make sure that full publication checks are run on the final version of your CIF prior to submission.

Publication of your CIF in other journals

Please refer to the *Notes for Authors* of the relevant journal for any special instructions relating to CIF submission.

PLATON version of 27/03/2017; check.def file version of 24/03/2017

Datablock Cd112K - ellipsoid plot

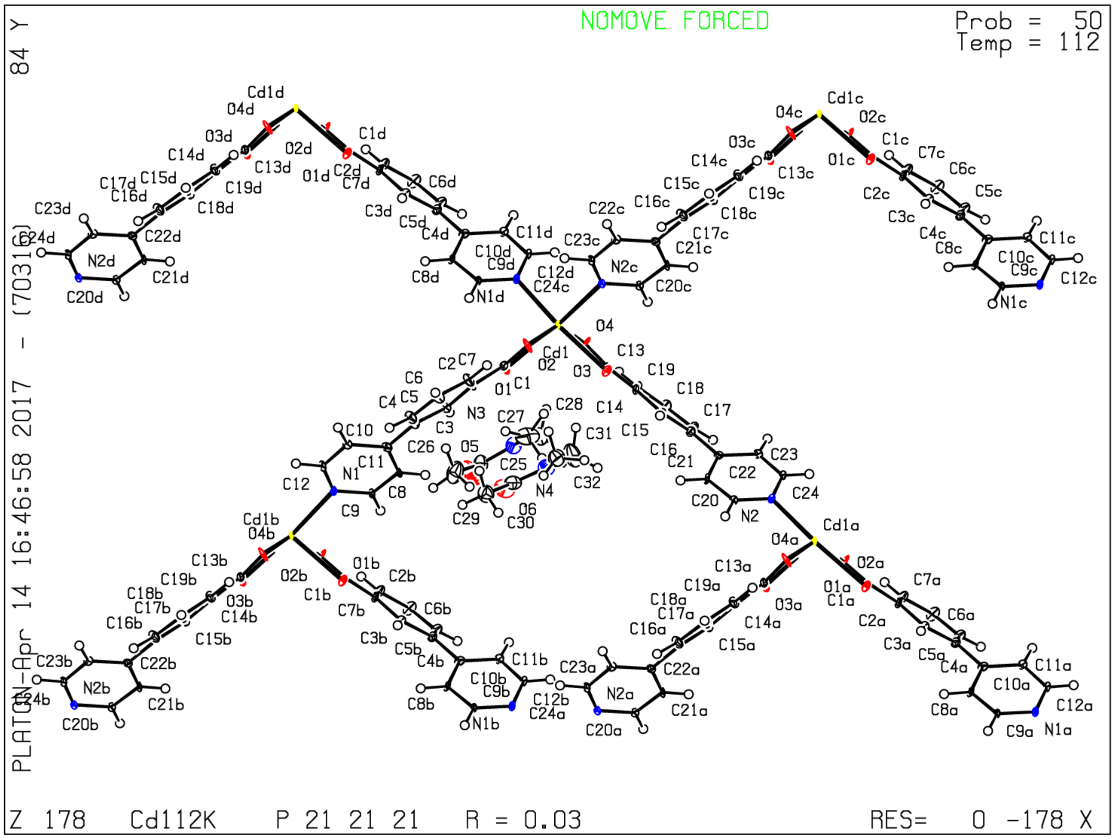

Supplement: Supplementary file 2 [file Data_Sheet_1.ZIP › Cd112Kcheckcif.pdf]
