# Supplementary material for: Controlling Thermal Expansion Behaviors of Fence-Like Metal-Organic Frameworks by Varying/Mixing Metal Ions
Source: Front Chem. 2018 Jul 24;6:306. doi: 10.3389/fchem.2018.00306 (PMC6066979; doi:10.3389/fchem.2018.00306)

checkCIF/PLATON report

Structure factors have been supplied for datablock(s) ZnCd300K

THIS REPORT IS FOR GUIDANCE ONLY. IF USED AS PART OF A REVIEW PROCEDURE FOR PUBLICATION, IT SHOULD NOT REPLACE THE EXPERTISE OF AN EXPERIENCED CRYSTALLOGRAPHIC REFEREE.

No syntax errors found. CIF dictionary Interpreting this report

Datablock: ZnCd300K

Bond precision: C-C = 0.0080 A Wavelength=1.54184

Cell:	a=11.9844(3)	b=15.5676(4)	c=17.2670(5)
	alpha=90	beta=90	gamma=90
Temperature:	300 K		

	Calculated	Reported
Volume	3221.48(15)	3221.47(15)
Space group	P 21 21 21	P 21 21 21
Hall group	P 2ac 2ab	P 2ac 2ab
Moiety formula	C24 H16 Cd0.25 N2 O4 Zn0.75, 2(C4 H9 N O)	C24 H16 Cd0.25 N2 O4 Zn0.75, 2(C4 H9 N O)
Sum formula	C32 H34 Cd0.25 N4 O6 Zn0.75	C32 H34 Cd0.25 N4 O6 Zn0.75
Mr	647.78	647.76
Dx,g cm-3	1.336	1.336
Z	4	4
Mu (mm-1)	2.546	2.546
F000	1346.0	1346.0
F000’	1345.24	
h,k,lmax	14,19,21	14,19,20
Nref	6520[3649]	4805
Tmin,Tmax	0.631,0.601	0.617,1.000
Tmin’	0.572	

Correction method= # Reported T Limits: Tmin=0.617 Tmax=1.000
AbsCorr = MULTI-SCAN

Data completeness= 1.32/0.74 Theta(max)= 73.802

R(reflections)= 0.0388(4229) wR2(reflections)= 0.1116(4805)

S = 1.033 Npar= 410

The following ALERTS were generated. Each ALERT has the format
test-name_ALERT_alert-type_alert-level.
Click on the hyperlinks for more details of the test.

Alert level B

```
PLAT915_ALERT_3_B No Flack x Check Done: Low Friedel Pair Coverage         46 %
```

Alert level C

```
PLAT241_ALERT_2_C High   'MainMol' Ueq as Compared to Neighbors of         O2 Check
PLAT241_ALERT_2_C High   'MainMol' Ueq as Compared to Neighbors of         O4 Check
PLAT241_ALERT_2_C High   'MainMol' Ueq as Compared to Neighbors of         C6 Check
PLAT243_ALERT_4_C High   'Solvent' Ueq as Compared to Neighbors of         O6 Check
PLAT243_ALERT_4_C High   'Solvent' Ueq as Compared to Neighbors of        C30 Check
PLAT243_ALERT_4_C High   'Solvent' Ueq as Compared to Neighbors of        C31 Check
PLAT243_ALERT_4_C High   'Solvent' Ueq as Compared to Neighbors of        C32 Check
PLAT244_ALERT_4_C Low    'Solvent' Ueq as Compared to Neighbors of         N3 Check
PLAT250_ALERT_2_C Large U3/U1 Ratio for Average U(i,j) Tensor ....        2.4 Note
PLAT412_ALERT_2_C Short Intra XH3 .. XHn     H26C   ..  H28A    ..       1.85 Ang.
PLAT911_ALERT_3_C Missing # FCF Refl Between THmin & STh/L=  0.600         33 Report
PLAT978_ALERT_2_C Number C-C Bonds with Positive Residual Density.          0 Note
```

Alert level G

```
PLAT002_ALERT_2_G Number of Distance or Angle Restraints on AtSite          6 Note
PLAT004_ALERT_5_G Polymeric Structure Found with Maximum Dimension          2 Info
PLAT012_ALERT_1_G No     _shelx_res_checksum found in CIF ........     Please Check
PLAT171_ALERT_4_G The CIF-Embedded .res File Contains EADP Records          1 Report
PLAT172_ALERT_4_G The CIF-Embedded .res File Contains DFIX Records          4 Report
PLAT232_ALERT_2_G Hirshfeld Test Diff (M-X)  Cd1    --  O2      ..       15.8 s.u.
PLAT232_ALERT_2_G Hirshfeld Test Diff (M-X)  Cd1    --  O4      ..       18.2 s.u.
PLAT232_ALERT_2_G Hirshfeld Test Diff (M-X)  Cd1    --  C1      ..        7.0 s.u.
PLAT232_ALERT_2_G Hirshfeld Test Diff (M-X)  Cd1    --  C13     ..        8.0 s.u.
PLAT232_ALERT_2_G Hirshfeld Test Diff (M-X)  Zn1    --  O2      ..       15.8 s.u.
PLAT232_ALERT_2_G Hirshfeld Test Diff (M-X)  Zn1    --  O4      ..       18.2 s.u.
PLAT300_ALERT_4_G Atom Site Occupancy of Cd1     is Constrained at       0.25 Check
PLAT300_ALERT_4_G Atom Site Occupancy of Zn1     is Constrained at       0.75 Check
PLAT301_ALERT_3_G Main Residue  Disorder .............(Resd  1)..          1 % Note
PLAT302_ALERT_4_G Anion/Solvent/Minor-Residue Disorder (Resd  2)..         33 % Note
PLAT779_ALERT_4_G Suspect or Irrelevant (Bond) Angle in CIF .... #         29 Check
              CD1  -N1   -ZN1     3.646   1.555   3.646             0.00 Deg.
PLAT779_ALERT_4_G Suspect or Irrelevant (Bond) Angle in CIF .... #         35 Check
              CD1  -N2   -ZN1     3.645   1.555   3.645             0.00 Deg.
PLAT860_ALERT_3_G Number of Least-Squares Restraints ............          4 Note
PLAT912_ALERT_4_G Missing # of FCF Reflections Above STh/L=  0.600        107 Note
```

```
   0 ALERT level A = Most likely a serious problem - resolve or explain
   1 ALERT level B = A potentially serious problem, consider carefully
  12 ALERT level C = Check. Ensure it is not caused by an omission or oversight
  19 ALERT level G = General information/check it is not something unexpected

   1 ALERT type 1 CIF construction/syntax error, inconsistent or missing data
  13 ALERT type 2 Indicator that the structure model may be wrong or deficient
   4 ALERT type 3 Indicator that the structure quality may be low
  13 ALERT type 4 Improvement, methodology, query or suggestion
   1 ALERT type 5 Informative message, check
```

It is advisable to attempt to resolve as many as possible of the alerts in all categories. Often the minor alerts point to easily fixed oversights, errors and omissions in your CIF or refinement strategy, so attention to these fine details can be worthwhile. In order to resolve some of the more serious problems it may be necessary to carry out additional measurements or structure refinements. However, the purpose of your study may justify the reported deviations and the more serious of these should normally be commented upon in the discussion or experimental section of a paper or in the "special_details" fields of the CIF. checkCIF was carefully designed to identify outliers and unusual parameters, but every test has its limitations and alerts that are not important in a particular case may appear. Conversely, the absence of alerts does not guarantee there are no aspects of the results needing attention. It is up to the individual to critically assess their own results and, if necessary, seek expert advice.

Publication of your CIF in IUCr journals

A basic structural check has been run on your CIF. These basic checks will be run on all CIFs submitted for publication in IUCr journals (*Acta Crystallographica*, *Journal of Applied Crystallography*, *Journal of Synchrotron Radiation*); however, if you intend to submit to *Acta Crystallographica Section C* or *E* or *IUCrData*, you should make sure that full publication checks are run on the final version of your CIF prior to submission.

Publication of your CIF in other journals

Please refer to the *Notes for Authors* of the relevant journal for any special instructions relating to CIF submission.

PLATON version of 27/03/2017; check.def file version of 24/03/2017

Datablock ZnCd300K - ellipsoid plot

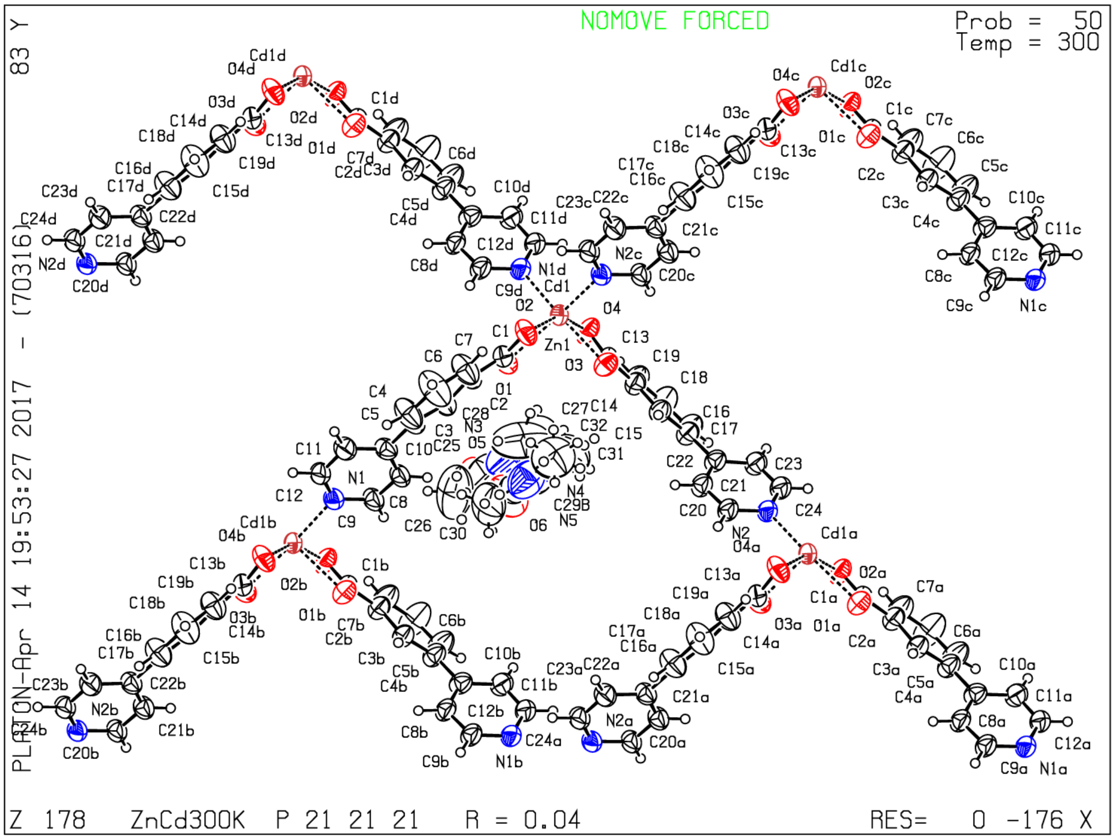

Supplement: Supplementary file 2 [file Data_Sheet_1.ZIP › ZnCd300Kcheckcif.pdf]
